# Childhood caries in the state of Kentucky, USA: a cross-sectional study

**DOI:** 10.1186/1472-6831-12-38

**Published:** 2012-09-05

**Authors:** Elizabeth A Kandel, Jenna M Richards, Catherine J Binkley

**Affiliations:** 1 School of Dentistry Pre-Doctoral Program, University of Louisville, Louisville, KY, USA; 2 Department of Surgical & Hospital Dentistry, School of Dentistry, University of Louisville, Louisville, KY, USA

## Abstract

**Background:**

Untreated dental caries afflicts almost one third of school-aged children in the United States and many of them are from disadvantaged families. This cross-sectional study was undertaken to investigate the prevalence of untreated caries in north central Kentucky, USA and to examine the relationships between the available demographic variables and untreated childhood caries as reported on the forms from the Smile Kentucky! program.

**Methods:**

During the fall of 2008, caries status was assessed during the visual oral screening examination component of “SmileKentucky!”– a model of the American Dental Association’s Give Kids A Smile program. Parents had completed brief surveys concerning 3,488 elementary school children aged 5 to 13 years who participated in the program. A secondary analysis was conducted using univariate, bivariate and multivariate statistical methods.

**Results:**

Untreated caries was reported in 33% of children. Bivariate and logistic regression analyses found that the most significant risk factors for having untreated caries were living in the metropolitan Louisville, Kentucky area, not having had a dental visit in the previous 3 years and not having any form of dental insurance.

**Conclusions:**

Untreated caries in elementary school children is prevalent in north-central Kentucky despite efforts to improve access to care. The results suggest that additional family and community preventive initiatives are needed to reduce the development of childhood caries in this area of the United States.

## Background

It has been demonstrated that dental care is the most prevalent unmet healthcare need among U.S. children [1,2] and especially among disadvantaged minority children [1,3]. Childhood tooth decay (i.e., caries) is the most powerful predictor of future experience of poor oral health [4]. Tooth decay that is left untreated can lead to pain, dysfunction, serious infections, and sometimes death [1,5].

National and state surveys have been conducted to determine untreated caries prevalence over the past 3 decades [2,6,7]. Tomar and colleagues recently reported that although some progress has been made in the oral health of our nation’s children, the objectives of Health People 2010 have largely not been met [8]. They reported that from 1988–1994 to 2004 untreated caries in children aged 6–8 years increased nationwide from 28% to 29%.

The recognition of oral health disparities in the United States and the need for public health surveillance led to the establishment of the National Oral Health Surveillance System (NOHSS) [9]. The system allows states to report 3 oral health indicators for 3^rd^ grade students who are generally 7–9 years old, including untreated caries, as often as their resources permit. Among the 35 states that participate in NOHSS, untreated caries in 3^rd^ graders ranges from 12% in New Hampshire to 43% in Texas. Kentucky’s last oral health screening was conducted in 2001 and as reported to NOHSS, 34.6% of children in the 3^rd^ grade had untreated caries – well above the 21% goal of Health People 2010 [10].

In an effort to increase access to dental care for poor children in north-central Kentucky, the university and dental society in Louisville partnered with the water company and Colgate to create the SmileKentucky! program [11]. The program is a model for the American Dental Association’s “Give Kids A Smile” annual volunteer initiative that provides preventive and restorative services to children from low-income families. In this program, “thousands of dentists across the country take time from their practices to help underserved children who aren’t getting the oral health care they need. Many dental hygienists, dental assistants, office managers and other volunteers help out too” [12].

SmileKentucky! has provided free treatment to over 2,800 uninsured children and free dental screenings to over 37,000 children since its inception in 2002. In the fall of each year, volunteer dentists and dental hygienists provide free dental screenings for approximately 3,500 children at elementary and middle schools across six counties in the north-central Kentucky area. The almost 100 volunteer dental professionals who conducted the screenings received an orientation on the program but were not calibrated for caries detection or assessment of treatment urgency. The screening information is used to identify children who need dental care but who have no public or private dental insurance. The parents of these uninsured children are invited to provide consent for the child to receive free transportation and treatment at the University of Louisville School of Dentistry a few months later in February [11].

Data has been collected since the program’s inception but it has not been analyzed and published. This cross-sectional study was undertaken to investigate the prevalence of untreated caries in north central Kentucky, USA and to examine the relationships between the available demographic variables and untreated childhood caries as reported on the forms from the Smile Kentucky! program.

## Methods

Data was extracted from the 3,488 parental demographic and consent forms and from the SmileKentucky! screening forms completed by dentists and/or dental hygienists in November 2008. Prior to the screening day, parents had completed a brief survey detailing the child’s gender, age, race/ethnicity, elementary school name, grade in school, home zip code as well as 7 questions regarding the child’s dental insurance status (government dental insurance, private dental insurance or no dental insurance), parental assessment of the condition of the child’s teeth (good, fair, or poor), health history, whether the child had a recent dental visit and the reason for the visit.

The dental screening form included yes (coded 1) or no (coded 0) responses for visible untreated caries, number of carious first molars (0 to 4), and number of quadrants with caries (0 to 4). Each child was then assigned a corresponding level of treatment urgency of no obvious problem, early stage dental disease, or urgent care needed.

Children were designated as residing in metropolitan or non-metropolitan areas by the zip code of residence reported by the parent. When the zip codes where the children resided were mapped, the metropolitan zip codes were located primarily within the city limits of Louisville, Kentucky area while the zip codes classified as non-metropolitan were suburban, small towns or rural areas to the east and south of the city. Metropolitan was coded as 1 and non-metropolitan was coded as 0.

Frequencies and bivariate chi-square and *t*-test analyses were used to examine the data for factors significantly associated with childhood caries. Regression analysis was used to examine factors for multicollinearity. Logistic regression analysis was used to explore which demographic factors increased the odds of childhood caries at the time of the study. *P* < 0.05 was considered statistically significant for all statistical tests. Data analysis was conducted with SPSS 20 (SPSS, Chicago, IL).

Parents or legally authorized representatives signed written consent for the oral screening examination and dental treatment. The study was approved by the University of Louisville’s Institutional Review Board.

## Results

The children ranged in age from 5 to 13 years with a mean of 9.3 years and their demographics are presented in Table 1. Parents reported that 92% of the children had visited a dentist in the past 3 years but upon examination by the dental screener, 33% were deemed to have caries present. The majority of children with caries had decay localized to a single quadrant and one quarter of children with caries were assessed as urgently needing dental treatment. Almost one-half of the children whose parents assessed their child’s oral health as fair or poor had visible caries. This was significantly different from the parents who rated their child’s oral health as good with one-quarter of the children having visible caries (*p ≤* 0.001).

**Table 1:** Participant characteristics (n = 3,488)

**Characteristic**	**n (%)**
**Gender**	
Male	1710 (49%)
Female	1777 (51%)
**Race/Ethnicity**	
White	2488 (71.3%)
Black	498 (14.3%)
Hispanic or Latino	143 (4.1%)
Asian	82 (2.4%)
American Indian	14 (0.4%)
Multiracial	154 (4.4%)
No Response	109 (3.1%)
**White or Minority Race/Ethnicity**	
White	2488 (73.6%)
Minority Race/Ethnicity	891 (26.4%)
**Area of Residence**	
Metropolitan	1579 (47.8%)
Non-metropolitan	1721 (52.2%)
Missing	188 (5.4%)
**Dental Insurance Coverage**	
Private Insurance	1830 (52.5%)
Government Insurance	1014 (29.1%)
No Insurance	584 (16.7%)
No response	60 (1.7%)
**Visit to Dentist in Past 3 Years**	
Yes	3216 (92%)
No	272 (8%)
**Reason for Last Dental Visit**	
Cleaning/Check-up	2739 (78.5%)
Dental Treatment	149 (4.3%)
Something was wrong	281 (8.1%)
Other	64 (1.8%)
Don’t know	44 (1.3%)
No Response	211 (6.1%)
**Presence of Caries**	
Caries Detected	1155 (33.1%)
No Caries Detected	2333 (66.9%)
**Parent Assessment**	
Good	2253 (64.6%)
Fair	1113 (32%)
Poor	108 (3%)
No Response	14 (0.4%)

We conducted bivariate analyses of demographic variables and parent assessment of the condition of their child’s teeth by untreated caries status. (Table 2). Of the 7 variables, 5 were found to be significant. These included being of minority race/ethnicity status, fair or poor parental assessment, having government or no insurance, not having a dental visit, and living in the metropolitan area. A fair or poor parental assessment was significantly correlated with the 5 significant factors and was not included as a variable in the multivariate analysis.

**Table 2:** Differences in children’s characteristics by untreated caries status

**Variable**	**No caries (n = 2333)**	**Caries (n = 1155)**	**Difference (P value)**
**Age – Mean (range)**	9.32 (5–13)	9.32 (6–13)	*p =* 0.95^Ŧ^
**Gender** (n = 3488)			
Male	1158 (50%)	552 (48%)	*p =* 0.456
Female	1175 (50%)	603 (52%)	χ^2^ (2df) = 1.57
**White Race vs Minority race/ethnicity** (n = 3379)*			
White	1730 (77%)	758 (68%)	*p ≤* 0.001
Minority race/ethnicity	532 (23%)	359 (32%)	χ^2^ (2df) = 28.62
**Area of Residence** (n = 3300)*			
Metropolitan	952 (57%)	627 (42%)	*p ≤* 0.001
Non-metropolitan	1277 (43%)	444 (58%)	χ^2^ (1df) = 72.68
Missing			
**Insurance Status** (n = 3428)*			
No insurance	356 (15%)	228 (20%)	*p ≤* 0.001
Govt or Private insurance	1949 (85%)	895 (80%)	χ^2^ (3df) = 74.86
**Has Visited Dentist** (n = 3488)			
Yes	2201 (94%)	1015 (88%)	*p ≤* 0.001
No	132 (6%)	140 (12%)	χ^2^ (1df) = 44.88
**Parent Assessment** (n = 3472)*			
Fair/Poor Oral Health	650 (28%)	571 (50%)	*p ≤* 0.001
Good Oral Health	1670 (72%)	581 (50%)	χ^2^ (1df) = 156.77
Missing			

* = some parent reported data missing.

Ŧ = Independent *t-* test.

χ^2^ = Chi-Square test.

df = degrees of freedom.

The finding that children residing in the metropolitan area were more likely to have untreated caries than those residing in a non-metropolitan area led us to conduct additional analyses because of the varied reports in the literature concerning caries prevalence in rural/non-metropolitan areas and metropolitan areas in the USA [7,13,14]. Caries was detected in 40% of the children from metropolitan areas and in 26% of the children from non-metropolitan areas (*p ≤* 0.001, χ^2^ (1df) = 72.680). Over one-third of metropolitan children with caries had caries in three or four quadrants, compared to less than one-fourth of non-metropolitan children with caries (*p ≤* 0.001, χ^2^ (4df) = 115.818). Twelve percent of the metropolitan children were assessed as urgently needing treatment, compared to only 5% of the non-metropolitan children (*p ≤* 0.001, χ^2^ (3df) = 31.103).

Forty-three percent of metropolitan children had private dental insurance compared to 63% of the children from non-metropolitan areas. Among the metropolitan children, 37% had government insurance compared to 22% of the non-metropolitan children. Similarly, 19% of the metropolitan children had no insurance at all compared to 22% of the non-metropolitan children. Seven percent of the metropolitan children had not visited the dentist in the previous 3 years, compared to 3% of non-metropolitan children.

We used logistic regression to explore factors associated with a child having caries and independent factors with *p* < 0.10 in the bivariate analysis were considered for the model. We first examined collinearity diagnostics using the methods recommended by Tabbachnick & Fiddel [15] in SPSS 20 and no evidence of multicollinearity was found. With childhood caries as the outcome, three independent demographic variables, living in the metropolitan area, no prior dental care in the previous 3 years and being uninsured, were retained in the model as significant using 0.05 as the selection criteria (Table 3).

**Table 3:** Untreated caries: final regression model

**Factor**	Estimate* (SD)	**Odds ratio**	**95% CI**
Metropolitan Area	0.578 (0.077)	1.78	(1.53-2.07)
Uninsured	0.234 (0.100)	1.26	(1.04–1.54)
No Dental Visit History	0.551 (0.142)	1.73	(1.31-2.28)

* Regression Coefficient or Beta weight.

SD, Standard Deviation; CI confidence interval.

## Discussion

This cross-sectional study was undertaken to investigate the prevalence of untreated caries in north central Kentucky, USA and to examine the relationships between the available demographic variables and untreated caries. One third of the children had untreated caries reported by the dentists who performed the oral screening examinations in the schools. Children were significantly more likely to have caries if they lived in the metropolitan area, had not seen a dentist for 3 years or were uninsured.

Our finding that children residing in a metropolitan area were more likely to have caries is consistent with results reported by Maserejian and colleagues who reported that children living in the metropolitan area of Boston, Massachusetts, USA had significantly more caries than children from the rural setting of Farmington, Maine, USA, even after controlling sociodemographic factors [13]. Similarly, Weyant and colleagues reported that children living in the metropolitan areas of Philadelphia and Pittsburg had the highest unfilled caries rate in permanent teeth than anywhere else in the state of Pennsylvania, USA [7].

Our finding differs from the North Carolina, USA study reported by Rozier and King who that found children residing in non-metropolitan areas adjacent to a metropolitan area to have higher rates of caries than children from metropolitan areas who had a lower caries rate [14]. Their data was obtained from the Behavioral Risk Factor Surveillance System (BRFSS) and the Child Health Assessment and Monitoring Program (CHAMP) which may have used a different definition of metropolitan and non-metropolitan which could account for the difference in findings.

The 33% overall caries prevalence in our study suggests that there may have been little change in the prevalence of childhood caries since the 2001 Kentucky survey for the National Oral Health Surveillance System (NOHSS) which reported 34.6% caries prevalence. Although the children in our study ranged in age from 5 to 13 years, the mean age was 9 years which is approximately the age of the children screened for the NOHSS. The results are concerning given the fact that there is a dental school located in downtown Louisville, Kentucky along with numerous private and public dental practices located in all the study areas. In addition, programs to improve access to dental care such as SmileKentucky! have been ongoing for almost a decade in this part of Kentucky.

The fact that only one-half of the uninsured children who had untreated caries noted during the screening examination later received dental treatment is also of concern. Smile Kentucky! eliminated structural obstacles to dental care by providing free transportation and free treatment in the University of Louisville pediatric dental clinic. Over 500 uninsured children were invited to receive treatment and parents provided consent for 350 of these children. On the SmileKentucky! treatment days in February 2009, only 236 uninsured children were transported and received comprehensive dental care in the pediatric dental clinic.

Anecdotal reports suggest that some parents did not want their child to be treated without them being present, the parents may have had poor functional literacy which prevented them from understanding the written invitation to receive free transportation and dental treatment, or they already had a dental home for their child. The reasons for failure to consent and/or failure to show up for dental care should be further investigated to determine what psychosocial factors may have prevented so many children from receiving treatment.

More focused preventive efforts may be required to reduce caries in disadvantaged children including home-based and/or school-based interventions. There is a clear need to reduce the development of childhood caries in addition to getting children to the dentist for cleanings, fillings and extractions [16]. Not only will this improve children’s oral health in the short and long term, but may also be more cost effective [17]. Public health policy makers may need to consider programs where the most at-risk children are identified and are provided preventive interventions at the family and/or community level.

There is some evidence that a dental care coordinator [18] can improve oral health outcomes for disadvantaged children. The American Dental Association supports the Community Dental Health Coordinator (CDHC) [19] pilot program which trains students from urban, rural and Native American communities to provide brief oral assessments, oral health education, preventive dental services and assistance in accessing and obtaining dental treatment. The CDHCs are community health workers with dental skills focusing on education and prevention and are “part social worker and part dental assistant who, under the supervision of a dentist, can help people navigate the public health system to get the dental care they need.” The CDHCs are trained to work in the community’s schools, clinics, senior citizen centers, Head Start Programs and other public health settings under the supervision of a dentist.

Attention may also need to be again directed at effective caries prevention initiatives including school based educational, [20] needs-related caries preventive, [21] sealant [22] and fluoride mouth rinse programs [23]. Improving nutrition in public schools by reducing carbohydrates in meals and vending machines may also lead to reduced caries and have the added benefit of helping to reduce childhood obesity [24]. Programs to educate pediatricians, primary care physicians and nurses in assessment of children’s oral health, counselling, referral for dental care, and application of fluoride varnish may also reduce caries development [25].

This study may have limitations due to the use of a convenience sample of children residing in counties in north-central Kentucky but the study’s large sample size of 3,488 children and data available for each child allowed for significant analyses of factors associated with untreated caries. The demographic information and dental history reported by parents was valuable but having more individualized information, such as actual household income, would have been beneficial. Another limitation was the lack of calibration of the almost 100 volunteer dental professionals who performed the oral screening exams. Although Smile Kentucky! is a community service program and calibration would have been difficult, future studies should include assessment of the reliability of the dental screening procedures across examiners. The study was also limited because the oral screening examinations were performed in the schools without the use of radiographs and as a result the untreated caries estimates may have been lower or higher than what truly existed [26,27].

## Conclusions

Untreated caries in elementary school children is prevalent in north-central Kentucky despite efforts to improve access to care. Children who resided in more densely populated metropolitan areas, children who were uninsured and children who had not seen a dentist in 3 years were more likely to have untreated caries. The results suggest that additional family and community level preventive interventions should to be evaluated for effectiveness in an effort to reduce the development of childhood caries and reduce oral health disparities.

## Competing interests

The authors declare that they have no competing interests.

## Authors’ contributions

EK and JR entered and cleaned the data. EK, JR and CB contributed scientifically to the paper by performing literature searches, data analysis and manuscript writing. All authors read and approved the final manuscript.

## Pre-publication history

The pre-publication history for this paper can be accessed here:

http://www.biomedcentral.com/1472-6831/12/38/prepub

## References

[B1] US Department of Health & Human Services Oral Health in America: A Report of the Surgeon General 2000 Bethesda, Maryland, USA: National Institute of Dental & Craniofacial Research http://www.nidcr.nih.gov/DataStatistics/SurgeonGeneral/sgr/home.htm

[B2] Edelstein Disparities in oral health and access to care: findings of national surveys Ambul Pediatr 2002 2 2 Suppl 141 147 11950385 10.1367/1539-4409(2002)002<0141:diohaa>2.0.co;2

[B3] Edelstein BL Chinn CH Update on disparities in oral health and access to dental care for America's children Acad Pediatr 2009 9 6 415 419 10.1016/j.acap.2009.09.010 19945076

[B4] Vargas CM Ronzio CR Disparities in early childhood caries BMC Oral Health 2006 6 Suppl 1 S3 10.1186/1472-6831-6-S1-S3 16934120 PMC2147596

[B5] ADA American Dental Association Statement on the Death of Deamonte Driver 2007 ADA Press Release [Press Release] March 2, 2007: http://www.ada.org/4198.aspx

[B6] Brown LJ Kaste LM Selwitz RH Furman LJ Dental caries and sealant usage in U.S. children, 1988–1991: selected findings from the Third National Health and Nutrition Examination Survey J Am Dent Assoc 1996 127 3 335 343 8819780 10.14219/jada.archive.1996.0203

[B7] Weyant RJ Manz M Corby P Dental caries status and need for dental treatment of Pennsylvania public school children in grades 1, 3, 9, and 11 J Public Health Dent 2004 64 3 136 144 10.1111/j.1752-7325.2004.tb02743.x 15341136

[B8] Tomar SL Reeves AF Changes in the oral health of US children and adolescents and dental public health infrastructure since the release of the Healthy People 2010 Objectives Acad Pediatr 2009 9 6 388 395 10.1016/j.acap.2009.09.018 19945073

[B9] Malvitz DM Barker LK Phipps KR Development and status of the National Oral Health Surveillance System Prev Chronic Dis. Apr 2009 6 2 A66 PMC2687872 19289009

[B10] CDC National Oral Health Surveillance System - Untreated Tooth Decay 2011 http://apps.nccd.cdc.gov/nohss/IndicatorV.asp?Indicator=3, 2011

[B11] SmileKentucky SmileKentucky 2011 http://www.smilekentucky.com/. Accessed June 14, 2011

[B12] ADA Give Kids a Smile 2011 http://www.ada.org/3452.aspx. Accessed June 10, 2011, 2011

[B13] Maserejian NN Tavares MA Hayes C Soncini JA Trachtenberg FL Rural and urban disparities in caries prevalence in children with unmet dental needs: the New England Children's Amalgam Trial J Public Health Dent 2008 68 1 7 13 10.1111/j.1752-7325.2007.00057.x 18179469

[B14] Rozier RG King RS Defining the need for dental care in North Carolina: contributions of public health surveillance of dental diseases and conditions N C Med J. Nov-Dec 2005 66 6 438 444 16438100

[B15] Tabachnick B Fidell L Using Multivariate Statistics 2001 4 Needham Heights MA: Allyn & Bacon

[B16] Shenkin JD An increase in caries rate or an increase in access to care: data show mixed results J Public Health Dent 2011 71 1 1 5 10.1111/j.1752-7325.2010.00198.x 20880028

[B17] Shenkin JD Patient-centered caries prevention in children may be more cost-effective in the long term than traditional dental care J Evid Based Dent Pract 2011 11 1 56 57 10.1016/j.jebdp.2010.11.004 21420017

[B18] Binkley CJ Garrett B Johnson KW Increasing dental care utilization by Medicaid-eligible children: a dental care coordinator intervention J Public Health Dent 2010 70 1 76 84 10.1111/j.1752-7325.2009.00146.x 19765202 PMC4060152

[B19] ADA Community Dental Health Coordinators 2011 http://www.ada.org/cdhc.aspx. Accessed August 28, 2011

[B20] Grocholewicz K The effect of selected prophylactic-educational programs on oral hygiene, periodontium and caries in school children during a 4-year observation Ann Acad Med Stetin 1999 45 265 283 10909495

[B21] Axelsson P The effect of a needs-related caries preventive program in children and young adults - results after 20 years BMC Oral Health 2006 6 Suppl 1 S7 10.1186/1472-6831-6-S1-S7 16934124 PMC2147598

[B22] Gooch BF Griffin SO Gray SK Preventing dental caries through school-based sealant programs: updated recommendations and reviews of evidence J Am Dent Assoc 2009 140 11 1356 1365 19884392 10.14219/jada.archive.2009.0070

[B23] Neko-Uwagawa Y Yoshihara A Miyazaki H Long-term caries preventive effects of a school-based fluoride mouth rinse program in adulthood Open Dent J 2011 5 24 28 10.2174/1874210601105010024 21566697 PMC3091371

[B24] Willershausen B Haas G Krummenauer F Hohenfellner K Relationship between high weight and caries frequency in German elementary school children Eur J Med Res 2004 9 8 400 404 15337630

[B25] Krol DM Children's oral health and the role of the pediatrician Curr Opin Pediatr 2010 22 6 804 808 10.1097/MOP.0b013e3283402e3b 20885329

[B26] Beltran ED Malvitz DM Eklund SA Validity of two methods for assessing oral health status of populations J Public Health Dent 1997 57 4 206 214 10.1111/j.1752-7325.1997.tb02977.x 9558624

[B27] Ketley CE Holt RD Visual and radiographic diagnosis of occlusal caries in first permanent molars and in second primary molars Br Dent J 1993 174 10 364 370 10.1038/sj.bdj.4808172 8494666

